# Activation of telomerase via madecassic acid enhances the developmental competence of the SCNT-derived bovine embryos

**DOI:** 10.3389/fcell.2025.1628953

**Published:** 2025-07-11

**Authors:** Zaheer Haider, Safeer Ullah, Tahir Muhammad, Chalani Dilshani Perera, Muhammad Tayyab Khan, Asif Jan, Seung-Eun Lee, Seo-Hyun Lee, Sung Woo Kim, Muhammad Idrees, Il-Keun Kong

**Affiliations:** ^1^ Division of Applied Life Science (BK21 Four), Gyeongsang National University, Jinju, Gyeongnam, Republic of Korea; ^2^ Molecular Neuropsychiatry & Development (MiND) Lab, Campbell Family Mental Health Research Institute, Centre for Addiction and Mental Health, Institute of Medical Science, University of Toronto, Toronto, ON, Canada; ^3^ Department of Pharmacy, University of Peshawar, Peshawar, Pakistan; ^4^ Hanwoo Research Institute, National Institute of Animal Science, Rural Development Administration, Pyeongchang, Gangwon, Republic of Korea; ^5^ Institute of Agriculture and Life Science, Gyeongsang National University, Jinju, Gyeongnam, Republic of Korea; ^6^ TheKingKong Corp. Ltd., Gyeongsang National University, Jinju, Gyeongnam, Republic of Korea

**Keywords:** SCNT, art, telomerase, *in vitro* embryo production (IVP), madecassic acid (MA)

## Abstract

Somatic cell nuclear transfer (SCNT) is important in assisted reproductive technologies. However, its reprogramming efficiency remains low. A considerable drawback of SCNT-cloned embryos is the reduction in telomerase activity, which is crucial for DNA stability and genetic and epigenetic reprogramming. The present study aimed to examine the effects of madecassic acid (MA), a potent telomerase activator, on the developmental rate, embryonic genome activation, and implantation potential of SCNT-derived bovine embryos. The treatment of bovine signal cell-cloned zygotes with 3.0 μg/mL MA significantly increased embryo cleavage (71.5%) and blastocyst rate (28.1%) compared with that in non-treated (control) SCNT-cloned bovine embryos. In addition, MA treatment enhanced the bovine granulosa cells' telomerase activity and telomerase expression are assessed using qTRAP assay and ELISA. Of note, MA enhanced the expression of embryonic genome activation (EGA)-related genes including *NFYA*, *SP1*, *DPRX*, *GSC*, *CTNNB1*, *DUX*, and *ARGFX* in MA-treated cloned embryos compared to the control group. Moreover, MA-treatment of cloned embryos showed substantially less DNA damage than the control SCNT embryos. Mechanistically, MA activation of telomerase reverse transcriptase (TERT) significantly enhanced the nuclear localization of β-catenin and c-Myc and improved EGA. Reduction in the nuclear localization of this triose may be the leading cause of reduced EGA in cloned embryos. In conclusion, MA impacted the EGA reprogramming and development of cloned bovine embryos via probable activation of TERT. This telomerase activator may have the application of improving SCNT-cloned bovine embryos.

## Introduction

Cloning is a technique applied to conserve mammalian species threatened with extinction and generate embryonic stem cells (ESCs) (Liu et al., 2016; Wilmut et al., 1997). Somatic cell nuclear transfer (SCNT) plays an important role in reproductive biotechnology; however, the efficiency of cloning remains low. To date, more than 20 animal species have been cloned by applying the SCNT cloning technology with the use of different cell types as nuclear donors (Czernik et al., 2019; Samiec, 2024). So far, cattle are the most common animals cloned, with elite bulls, high-milking cows, genetically modified specimens and endangered or critically endangered breeds having been successfully generated (Akagi et al., 2013; Skrzyszowska and Samiec, 2021). The development of human nuclear transfer derived stem cells from patients and older adult donors holds promise for future therapeutics (Li et al., 2019; Saito et al., 2019; Yang et al., 2007). The SCNT-based cloning has several drawbacks, such as a very low success rate, aberrant reprogramming of somatic cell-inherited epigenomic landscapes, developmental impairments, and premature aging observed in nuclear-transferred embryos, conceptuses and progeny (Samiec et al., 2019; Samiec and Trzcińska, 2024). To increase the efficiency of somatic cell cloning, it is indispensable to identify and thoroughly evaluate a wide spectrum of factors having impacts on the developmental capability and structural-functional quality of mammalian intra- and interspecies SCNT-derived embryos, including their bovine representatives (Alsalim et al., 2019; Opiela et al., 2017; Samiec, 2022). Among the above-indicated factors, the source of nuclear donor cells and reprogrammability of donor cell nuclei are pivotal determinants affecting SCNT effectiveness (Saini et al., 2015; Samiec and Skrzyszowska, 2010). Moreover, an important role is exerted by the qualitative parameters related to interplay between meiotic, cytoplasmic and epigenomic maturation of nuclear recipient oocytes as well as their abilities to artificially activate embryogenesis (Choi et al., 2013; Keim et al., 2023; Pan et al., 2015). Finally, the molecular interaction between nuclear and mitochondrial DNA fractions considerably affect the efficiency of producing SCNT-derived embryos (Hiendleder, 2007; Samiec, 2005; Srirattana et al., 2011).

Telomerase reverse transcriptase (TERT) is the main enzyme that elongates telomeres by adding a unique sequence (TTAGGG) to their ends (Wu et al., 2017). Telomere activities include chromosomal segregation, replication, and stability. With the addition of telomere sequence, the chromosome is protected from end-to-end fusion, degradation, and recombination (Blackburn, 1991; Doksani et al., 2013; J. H. Teichroeb et al., 2016). The role of telomerase begins with the onset of animal growth, as telomerase activation plays a role in embryonic genome activation (EGA) during the 8- to 16-cell stage of embryo (Zhai et al., 2022). A significant drawback of SCNT is telomere shortening or silencing of telomerase, as identified by Shiels et al. (Kurome et al., 2008; Shiels et al., 1999). The first cloned animal, Dolly, had a shorter telomere length than the controls of the same age (Burgstaller and Brem, 2017). Telomere is a key marker for the molecular aging of cells (Schaetzlein and Rudolph, 2005). Telomerase activation significantly affects several genes essential for embryonic development. Several critical processes and molecules are important for embryonic development, including the Wnt/beta-catenin pathway, c-Myc, a pluripotency gene, and p300, a protein involved in transferring an acetyl group to histones, which interacts directly or indirectly with telomerase (Antolín et al., 1996; Vervoorts et al., 2003; Zhang and Zhang, 2014). Furthermore, telomere length and telomerase activity influence the regulation of mitochondrial fitness (Zheng et al., 2019).

Recently, telomerase activation by numerous natural drugs is gaining support as a potential treatment or prevention of several degenerative diseases (Harley et al., 2013; Tsoukalas et al., 2019). Madecassic acid (MA) is a natural compound isolated from the *Centella asiatica* extract formulation 08AGTLF. Both 08AGTLF and its active compound MA, have been demonstrated to have powerful telomerase-enhancing activity (Tsatsakis et al., 2023; Tsoukalas et al., 2019) and potential antioxidant properties (Bertollo et al., 2024). The present study aimed to evaluate the effect of MA on the development of the SCNT-cloned embryos. Our data revealed that MA exposure impacted the EGA reprogramming and improved the developmental potential, epigenetic modifications, and the implantation competence of the bovine SCNT-cloned embryo via probable activation of TERT.

## Materials and methods

Unless specified, Sigma Aldrich (St. Louis, MO, United States) was the source of all chemicals and reagents. Every experimental method was carried out strictly in compliance with the guidelines set out by the Institute of Animal Care Committee at Gyeongsang National University (GNU-230425-A0088).

### Experimental design

Madecassic acid dissolved in dimethyl sulfoxide (DMSO), was diluted in phosphate-buffered saline (PBS) and at different concentrations (1–5 μg/mL) and was added to 0.5 mL of invitro-culture media (IVC_1_) and blastocyst development was analyzed. The effective concentration was selected based on blastocyst development, using five biological replicates containing 40 embryos. Protein levels were analyzed using immunofluorescence. RT-qPCR was used to analyze the mRNA levels of many genes associated with epigenetics, stem cells, and mitochondria in 8-cell stage embryos and blastocysts.

### Reagents and antibodies

Madecassic acid (MA) was purchased (99% purity) from ApexBt (Cat. #N2355). The mRNA was extracted through picopure RNA extraction kit (Arcturus, Thermo Fisher Scientific; KIT0204) and cDNA were made by using superscript III reverse transcriptase (Bio-rad laboratories, Hercules, CA, United States; Cat # 1708891). Cell permeability was measured using Tritirachium album proteinase K (Thermo Fisher Scientific; CAS No. 39450-01–6). The primers that were used were from Macrogen, Inc. The Santa Cruze Biotechnology in Dallas, Texas, United States, provided the antibodies against *TERT* (Cat. # sc-393013), H3K9ac (Cat. # MA5-11195), 5 mC (Cat. # MA5-31475), C-Myc (Cat. # MA1-980), CDX-2 (Cat. # MA5-35215), and stat3 (Cat. # sc-5279 AF546). 2′,7′-dichlorodihydrofluorescein diacetate (H2DCFDA) from Sigma Aldrich (Cat. #D6883) was used to evaluate the amounts of reactive oxygen species (ROS). Cell death detection kit TMR red (label solution and enzyme solution; Sigma Aldrich, St. Louis, MO, United States; Cat. #12156792910) was utilized *in situ* at a 1:9 dilution. The telomerase activity was analyzed using ELISA (Elabsciences, Hoston, TX, United States; Cat. #E-ELM1125).

### Collection and maturation of COCs

IVM (*in vitro* maturation) was carried out as previously explained (Khan et al., 2023). Bovine ovaries from the Hanwoo cow ovaries were collected from a local slaughterhouse within 2 hours of slaughter and transported to the lab in sterile saline at 35°C. To remove the cumulus oocytes complex from the follicles (2–8 mm in diameter), an 18-gauge needle attached to the vacuum pump is used following ovulation washing with fresh D-PBS. A Petri plate containing the aspirated COCs was supplemented with the Tyrode lactate-HEPES (TL-HEPES) medium (2 mM Na2CO3, 114 nM NaCl, 0.5 mM MgCl_2_, 1 μL/mL phenol red, 10 mM HEPES, 0.1 mg/mL streptomycin, and 100 IU/mL penicillin). The COCs were collected using a stereomicroscope. Following a TL-HEPES medium wash, COCs with uniform cytoplasm and compacted cumulus layers were incubated in 700 µL of the IVM medium containing TCM-199 supplemented with 8% fetal bovine serum (FBS), 10 μg/mL follicle-stimulating hormone, 1 μg/mL estradiol-17β, 10 ng/mL epidermal growth factor, 0.6 mM cysteine, and 0.2 mM Na-pyruvate for each group. For 22–24 h, the plates were incubated at 38.5°C with 5% CO_2_.

### *In vitro* Fertilization

*In vitro* fertilization (IVF) was carried out as mention previously (Ullah et al., 2025) Click or tap here to enter text. In brief, cryopreserved sperm from Hanwoo bulls were used to fertilize mature oocytes. The frozen sperm were thawed in water at about 37.5°C for 30 , and then they were rinsed with sperm-distilled PBS. For five min the pellet is centrifuged at 750 × g to collect the sperm pellet. Next, 100 IU/mL penicillin, 0.1 mg/mL streptomycin, 22 mg/mL sodium pyruvate, and 6 mg/mL bovine serum albumin (BSA) were added to the Tyrode lactate solution. The sperm pellets were then resuspended using 500 µL of heparin (20 mg/mL), and this procedure was repeated. The sperm solution was incubated for a further 15 min at 38.5°C with 5% CO_2_. Prior to microscopic inspection, the concentration of 1 × 10^6^ spermatozoa/mL is diluted. The oocytes were incubated for 10 to 18 h in 4-well plate containing prepared 0.5 mL IVF media at 38.5°C with 5% CO_2_.

### Preparation of donor cells

After being cleaned with DPBS, the skin tissue of Hanwoo cows was treated with a cell washing solution that contained 0.20% (v/v) trypsin-EDTA (Gibco BRL, Life Technologies, Grand Island, NY, United States). The cells were centrifuged at 1000 rpm, and cultured in Dulbecco’s modified Eagle’s medium (DMEM; Gibco BRL, Life Technologies, Grand Island, NY, United States) supplemented with 15% FBS, 1% penicillin, 1% L-glutamine, and 1% nonessential amino acids. While doing the second passage some of the primary cells were cryopreserved for later use as Donar cells in 90% FBS and 10% DMSO.

### Somatic cell nuclear transfer

After incubation in the IVM medium for 22–24 h, cumulus cells from the COCs were separated by gentle pipetting in a hyaluronidase solution containing 0.1% testicular hyaluronidase produced in TL-HEPES. The initial polar bodies from the denuded oocytes were enucleated as described previously (Xu et al., 2019). To finish the enucleation process, the first polar body and a tiny quantity of surrounding cytoplasm were aspirated in TCM-199 medium microdrops containing 0.3% BSA and 7.5 μg/mL cytochalasin B (CB). As previously mentioned, 80% confluent somatic cells were separated and submerged in the Sendai virus (SV; Cosmo Bio, Tokyo, Japan) solution for 1 min (Song et al., 2011). Briefly, 260 µL of suspension buffer was combined with the inactivated, freeze-dried SV envelope, and the mixture was further diluted with fusion buffer at a ratio of 1:4. Donor cells were injected to the enucleated oocytes' perivitelline region (Imsoonthornruksa et al., 2012). After the SV-mediated fusion of the reconstructed embryos had been terminated, they were incubated for 2 h in the synthetic oviductal fluid (SOF) medium enriched with BSA, insulin-transferrin-sodium selenite (ITS) solution, epidermal growth factor (EGF) and additionally supplemented with 5 μg/mL CB. To activate the successfully reconstructed embryos, they were incubated in 5 μM calcium ionomycin for 5 min. In the next step, SCNT-derived embryos were incubated in 2 mM 6-dimethylaminopurine (6-DMAP) in a humidified environment at 38.5°C under 5% CO_2_ for 4 h.

### *In vitro* culture

The cloned embryos were cultured as described previously (Mesalam et al., 2019). After the activation, the cloned embryos were washed in 600 µL SOF + BSA + EGF + ITS and keep it for 3 days in 38°C with 5% CO_2_ in humidified environment in 4-well plate (NUNC; Roskilde, Denmark). After the eight-cell stage, the cloned embryo was cultured until day 8 blastocyst stage (day 0 = fusion). On day 8, blastocyst growth was confirmed by observing under a stereomicroscope.

### Telomerase activity assay

Before being treated with MA, bovine ovarian granulosa cells were cultivated for 24 h in cell culture media that contained 1% penicillin, 15% v/v FBS, and DMEM. After treating the cells with MA for 24 h, the proteins were isolated from the cells by sonication. A colorimetric sandwich ELISA (LS-F12714) was used to quantitatively assess the amounts of bovine telomerase in the plasma samples. At 450 ± 2 nm, the optical density was observed.

### qTRAP assay

qTRAP is performed as previously mention (Kazemi Noureini et al., 2018). The bovine granulosa cells were cultured in six well plates and treated 3 μg/mL of Madecassic acid for. The cells were trypsinized and washed with cold PBS. For 30 min, the cells were treated in lysis buffer that contained 10 mM Tris–HCl, pH = 7.5, 1 mM MgCl2, 1 mM EGTA, 0.1 mM phenylmethylsulfonylfluoride (PMSF), 5 mM beta-mercaptoethanol, 0.5% CHAPS, and 10% glycerol. After 30 min of centrifugation at 14,000 x g, the supernatant was collected, and the Bradford test was used to determine the protein content. The q-TRAP reaction mixture had a total volume of 20 μL and contained 10 μL of SYBRGreen Kit (Bio-Rad), 10 pM primer TS 5-AATCCGTCGAGCAGAGTT-3 ´, and H2O (DEPC). The reaction mixture was incubated for 20 min at 25 ˊC. 5 pM ACX 5 ´-GCGCGG (CTTACC)3CTAACC-3 ´ was then added, and real-time PCR was carried out using the cycling profile: 40 cycles of 30 s at 94°C, 30 s at 50°C, and 45 s at 72°C; one cycle of 10 min at 95°C.

### Determination of relative telomere length

As explained by Cawthon (Cawthon, 2002) a real-time quantitative PCR (qPCR) technique was used to measure relative telomere length (RTL). ZAR1 was used as a single-copy reference gene to measure expression using modified telomere primers (Cawthon, 2009). The telomere primers telg, 5′-ACACTAAGGTTTGGGTTTGGGTTTGGGTTTGGGTTAGTGT-3′ and telc, 5′-TGTTAGGTATCCCTATCCCTATCCCTATCCCTATCCCTAACA-3′ produce a single fixed-length product. 5′-AAGTGCCTATGTGTGGTGTG-3′ was the ZAR1 forward primer sequence, and 5′-CAGGTGATATCCTCCACTCG-3′ was the reverse primer sequence (Macrogen). Quantitative PCR was performed in real time using a Bio-Rad CFX96TM (Bio-Rad) device. DNA from embryo lysate, 0.5 μm primer mix (final concentration of each primer), 5 μL of 2× SsoFast EvaGreen supermix (Bio-Rad), and DNase-free water were the final reagent volume and concentrations in the PCRs, resulting in a final reaction volume of 10 μL.

The thermal cycler profile used to determine telomere length was as follows: 45 cycles of 15 s at 94°C, 10 s at 62°C, and 15 s at 74°C with a signal acquisition, followed by melt curve acquisition cycles; one cycle of 15 min at 95°C; two cycles of 15 s at 94°C, 15 s at 49°C. One cycle of 3 min at 98°C, forty-five cycles of 10 s at 95°C and 60 s at 60°C with signal capture, and melt curve analysis comprised the reference gene profile. The T/S ratio of the telomere product amplification (T) to the single-copy reference gene (S) was used to calculate the relative telomere length. Telomere length was reported in relation to the reference after each sample was tested three times.

### mRNA extraction and RT-qPCR

RT-qPCR was performed as described previously (Idrees et al., 2025; Idrees et al., 2021). Total mRNA was isolated from day 8 cells and day 8 blastocyst using an Arcturus PicoPure RNA Isolation Kit (Arcturus, Foster City, CA, United States), following the manufacturer’s instructions. The concentration of the purified mRNA was measured at 260 nm using a Nanodrop 2000c spectrophotometer. The first cDNA strand was generated using the Bio-Rad Laboratories Script cDNA Synthesis Kit (Hercules, California, United States). Until needed for quantitative reverse transcription PCR (RT-qPCR), the produced cDNA was stored at −80°C. Table 1 lists the sequences of primers and PCR parameters used for each gene.

**TABLE 1:** *Primer sequences used in RT-qPCR*.

Genes	Primer sequence	Product size (bp)
GAPDH	5′-CCCAGAATATCATCCCTGCT 3′ 5′-CTGCTTCACCACCTTCTTGA-3′	185
*TERT*	5’ -CGGACAGCCCGAGCAC-3‘ 5’ -GGTCTTGAAGTCTGCGGTCA-3′	99
β-Catenin	5’ -AATCAGCTGGCCTGGTTTGA-3′ 5′-GCTTGGTTAGTGTGTCAGGC-3′	145
OCT4	5’ -CCACCCTGCAGCAAATTAGC3′ 5′-CCACACTCGGACCACGTCTT-3′	68
iNOS	5′CGAGCTTCTAACCTCAAGCTATC-3′ 5′-CTGGCCAGATGTTCCTCTATTT-3′	172
CDX-2	5′GCAAAGGAAAGGAAAATCAACAA-3′ 5′-GGCTCTGGGACGCTTCT-3′	82
NANOG	5′-CCAGGGGTGTTTGGTGAACT-3′ 5′-TGCTCCACGTGGGGTTATTC-3′	74
Klf-4	5′-CTCGGGCAATTTGGGGTTTG-3′ 5′- CCAAAAGGTCCTCGGGAGTC-3′	111
SOX-2	5′-CGCCCTGCAGTACAACTCTA-3′ 5′-GGGTGCCCTGCTGAGAATAG-3′	89
p300	5′-CTGGTGGAGGAATGCCCAAT-3′ 5′-AGCTGTATGTGCCCCAGAAC-3′	107
c-Myc	5′-CCA GTA GCG ACT CTG AGG AAG-3′ 5′-TGT GAG GAG GTT TGC TGT GG-3′	117
DUXA	5′-GCCGTACCTCGTTCACAGAA-3’ 5′-GCCGTACCTCGTTCACAGAA-3′	70
DPRX	5′-GCGTCCAGACTTGCACAAAG-3′ 5′-TGCCAACTGTTTCTCCGTGA-3′	169
ARGFX	5′-GCTAGTGGCCTCAGTTCCTG-3’ 5′-GGAGGTGGTCACATAACGCA-3′	73
NFYA	5′-GATTTGGAGGGGCCATGGAA-3′ 5′-CATTAATGGCTGCCCCTGGA-3′	197
SP1	5′-TGCTACCATGAGCGACCAAG-3′ 5′-CAAAGGGGATGGCTGGGATT-3′	190
GSC	5′-GACCAAGTACCCAGACGTGG-3′ 5′-TCTCAGCGTTTTCCGACTCC-3′	96
BRG-1	5′-TCGTGAGAAGAAGCGAGACG-3′ 5′-ACATCTTCACAGGAGCTGCG-3′	124
Tet-3	5′-AGTTCCAGACAGAATGCGGG-3′ 3′-CACCACTGGGCTGAAGCTAA-3′	148
PGC-1	5′-GATTAGTTGAGCCCTTGCCG-3′ 5’ -GCCAGGAGTTTGGTTGTGAT-3′	163
BAX	5′-CACCAAGAAGCTGAGCGAGTGT-3′ 5′-TCGGAAAAAGACCTCTCGGGGA-3′	118
BCL2	5′-TGGATGACCGAGTACCTGAA-3′ 5′-CAGCCAGGAGAAATCAAACA-3′	120

### Immunofluorescence staining

The immunofluorescence staining procedure was carried out as previously mentioned (Ullah et al., 2025). In brief, the embryos were preserved at 4°C for 30 min after being fixed in a 4% formaldehyde solution at room temperature. They were then rinsed three times for 10 min each in PVA-PBS (0.3%). The antigens were then recovered by adding proteinase K for 5 minutes. After two rounds of washing, the blastocysts were incubated in the blocking solution (5% BSA) for 90 min. Primary antibodies against 5 mC and acH3K9 were incubated with 8-cell embryos, whereas blastocysts were incubated with antibodies against CDx-2, C-Myc, Oct-4, State3, and *TERT* with 0.25% Triton X-100 at 4°C. The incubation was carried out overnight. Following three 15-min PVA-PBS washes, the samples were incubated for 90 min at room temperature with secondary antibodies that were FITC- and TRITC-tagged (Santa Cruz Biotechnology, Dallas, TX, United States). The samples were incubated with 10 μg/mL DAPI for 5 minutes following three PVA-PBS washes. Two PVA-PBS washes lasting 10 minutes each came next. A confocal laser-scanning microscope (Fluoview FV 3000; Olympus, Tokyo, Japan) was used to analyze the samples after being fixed on a glass slide. The signal intensity was analyzed using ImageJ software (National Institutes of Health, Bethesda, MD, United States).

### Terminal deoxynucleotidyl transferase dUTP nick end labeling assay

The assay was carried out as previously reported (Khan et al., 2023). In brief, preserved blastocysts were stained using the *in-situ* cell detection kit TNR (red label solution and enzyme solution) (Sigma Aldrich, St. Louis, MO, United States; Cat. #12156792910) at a 1:9 dilution after being washed three times in PVA-PBS. The stained blastocysts were incubated for 1 h at 38.5°C in 5% CO_2_. After three PVA-PBS washes, the blastocysts were stained for 5 min with DAPI. An epifluorescence microscope (Olympus IX71; Olympus, Tokyo, Japan), equipped with a mercury lamp, was used to examine the dyed blastocysts. The relative integrated signal density was analyzed through imagJ software, and the dead cells are TUNEL-positive cells (red signals).

### H2DCFDA ROS assay

ROS levels in blastocysts were measured using the H2DCFDA ROS Detection Kit (Cat. # 6883). The blastocyst was incubated for 30 min with 10 nM H2DCFDA at 38.5°C in 5% CO_2_. The blastocysts were washed three times with PBS and observed under an epifluorescence microscope.

### Implantation assay

The implantation assay was conducted as described previously (Perera et al., 2023) to evaluate the 8-day blastocysts' invasive area and rate of growth. The cell inserts (6.4 mm; Corning Inc., Corning, NY, United States) were put into a 24-well plate for this experiment. After applying Matrigel (20 mg/filter; Discovery Labware Inc.) to the chamber’s upper surface, it was allowed to dry for half an hour. After that, the blastocysts were moved to filters coated with Matrigel. In the same SOF medium used to develop the embryos, each culture insert had three blastocysts. They were cultured for 72 h in a humidified environment at 38.5°C with 5% CO_2_, changing media every 48 h. After 74 h, the inner cell mass (ICM) and trophectoderm cells attached to the surface, start to proliferate, and show spreading behavior, all of which suggested implantation-like activity. For 5 minutes, the cells were labeled with DAPI. An Olympus IX71 microscope was used to measure trophoblast invasion and proliferation after a 10-day culture period. ImageJ program (version 154) to analyze the data.

### Protein preparation and molecular docking

The structure of bovine telomerase reverse transcriptase (bTERT) was downloaded from the AlphaFold protein structure database (https://www.alphafold.ebi.ac.uk/, ID: AF-A0A3Q1M466-F1-v4) and used as a receptor for docking with previously reported cycloastragenol (used as a reference) and MA as a test ligand. QuickPrep, a program for the Molecular Operating Environment (MOE) 2022.02, was used to import the protein for further preparation (Bakim and Jamaluddin, 2022). For further processing, the protein was imported into the QuickPrep tool of the Molecular Operating Environment (MOE) 2022.02 (Inc, 2016). It involved adding hydrogen atoms and allocating charges according to the force field of OPLS-AA molecular mechanics. The active sites for the bTERT protein were defined using the MOE built-in site finder, and the top five ranked sites were selected. Induced-fit docking was performed using the default parameters.

### Molecular dynamics (MD) simulation

To learn more about the stability, interaction dynamics, ligand characteristics, and molecular dynamics (Bakim and Jamaluddin, 2022) simulation was initiated using Schrödinger’s Desmond software (Yuan et al., 2016). An OPLS3e force field was used to decrease the energy of the system. The compounds were enclosed in a 10 Å orthorhombic box using a TIP3P water solvent model. The simulation was run for 100 ns, producing 1000 frames, with the temperature stabilizing at 300 K and the pressure maintained at 1 bar after the injection of ions and system neutralization. The production phase was prolonged by 50 ns to allow system relaxation after minimization. The Simulation Interaction Diagram tool in Schrödinger’s Maestro was used to view and evaluate the output data, which included ligand properties, root-mean-square deviation (RMSD), and root-mean-square fluctuation (RMSF).

### Statistical analysis

GraphPad Prism 6 (GraphPad Software, San Diego, CA, United States) was used for all statistical analyses. Embryo development was examined using a t-test, and a P-value of less than 0.05 (p < 0.05) was deemed statistically significant. Immunofluorescence density and integral optical density were analyzed using GraphPad Prism and ImageJ.

## Results

### Optimal concentration of madecassic acid (MA) and its effect on embryonic development and telomerase activity

**FIGURE 1:**
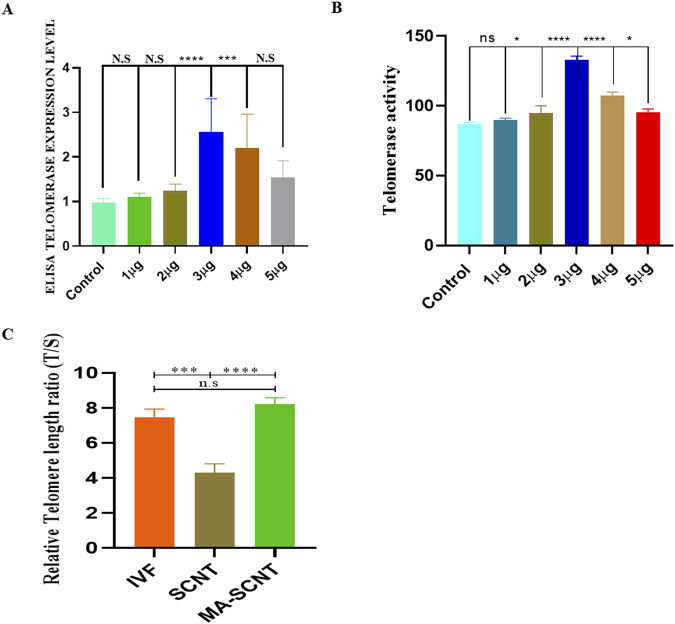
Optimal concentration of MA and telomerase activity in bovine granulosa cell. **(A,B)**The figure shows telomerase activity via qTRAP assay and TERT expression of the MA assessed via immune sorbent assay (ELISA) in the bovine granulosa cells. The cells were exposed to different concentrations of MA (1–5 μg/mL). Control cells were only grown in maintenance media. **(C)**The figure show the telomere length in IVF, SCNT and MA treated SCNT embryos. Three biological replicates were used. ns is not significant. Significant differences are indicated by ****p < 0.0001, ***p < 0.001. Standard error of the mean SEM.

To investigate the optimum concentration of MA effective for bovine embryo development, the *in vitro* fertilization (IVF) was performed, and the embryos were treated with various concentrations (1–5 μg/mL) of MA in the *in vitro* embryo culture (IVC) medium (Supplementary Table S1). In our experimental condition, treatment with 1, 2, 4, and 5 μg/mL of MA resulted in no significant effect on blastocyst development (28.9%, 29%, 30%, and 27.9%, respectively) as compared to the non-treated control group (28.5%). However, the IVF-derived embryos treated with 3.0 μg/mL of MA exhibited a considerably higher developmental capability to reach the blastocyst stage (37.5%) than the control non-treated IVF embryos (28.5%) (Supplementary Table S1). Similarly, SCNT-derived embryos exposed to 3.0 μg/mL of MA (MA-SCNT group) displayed a significantly improved progressive cleavage activity (71.5%) and blastocyst formation rate (28.1%) compared with that in the control non-treated SCNT embryos (Con-SCNT group) (Table 2). In addition, as assessed using ELISA, a significantly higher telomerase expression was observed in bovine granulosa cells at an MA concentration of 3.0 μg/mL compared to control and other treated groups (Figure 1A). level. While in qTRAP assay, the MA treatment to bovine granulosa cells at the concentration 3.0 μg/mL showed highly significant telomerase activity. (Figure 1B). Additionally, the qPCR analysis revealed that the SCNT group has significantly shortened telomere length compared to IVF and MA-SCNT group. However, the MA-treated SCNT had no significant difference in telomere length compared to IVF (Figure 1C). Given its significant effects, the optimized concentration of 3.0 μg/mL was selected for further experiments throughout the study.

**TABLE 2:** Determining the effect of the optimal MA concentration on the developmental outcomes of bovine cloned embryos.

Optimal concentration	No. of oocytes	No. of fused NDC-OCs (%)	No. of 2-cell embryos (%) 1	No. of 8-cell embryos (%) 2	No. of blastocysts (%)
Control SCNT	450	300 (66.6)^a^	271 (90.3 ± 1.01)^b^	191 (63.6 ± 1.17)^b^	63 (21.0 ± 1.32)^b^
3 μg/mL MA-SCNT	500	334 (66.8)^a^	305 (91.3 ± 1.32)^a^	239 (71.5 ± 1.72)^a^	94 (28.1 ± 1.41)^a^

NDC-OCs, nuclear donor cell-ooplast complexes; one early cleavage activity; two progressive cleavage activity (ability to reach the early morula stage); a, b denoting the significance of the differences between both control and experimental groups of fused NDC-OCs, as well as control and experimental groups of 2-cell embryos to the superscripts denoting the lack of significant differences a, a.

### Effect of MA on embryonic genome activation (EGA) and epigenetic modification in SCNT-Derived embryos

**FIGURE 2:**
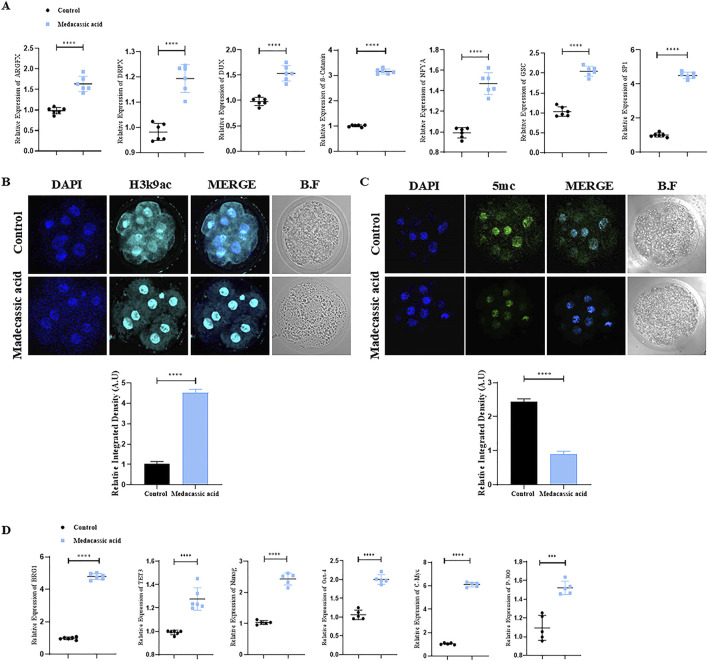
Effect of MA on zygotic genome activation and epigenetic modification in bovine cloned embryos. **(A)** mRNA expression of the genes ARGFX, DRPX, CUX, β-catenin, NFYA, GSC, and SP-1 were analyzed in the control and treated group using the PCR. **(B,C)** Immunofluorescence labeling was used to measure the amounts of H3K9ac and 5 mC proteins in the control and MA group. FITC (green), Alexa Flour 647 (cyan). **(D)** mRNA expression of the genes BRG1, TET3, Nanog, Oct-4, C-Myc, and P-300 were analyzed in the control and MA treated group using the PCR. The mean ± SEM is used to display the data. The ****p ≤ 0.0001 indicate significant differences. BF is for bright fields. Standard error of the mean is referred to as SEM.

EGA plays a crucial role in mammalian embryonic development (Hu et al., 2019). We observed an increase in the relative mRNA expression of genes in cloned bovine embryos, associated with EGA, such as NFYA, SP1, DPRX, GSC, ß-catenin, DUX, and ARGFX in the MA-SCNT group compared to the control group (Figure 2A). Epigenetic modifications are essential for normal embryonic development as they regulate gene expression patterns that determine cellular identity and differentiation (Du et al., 2022). Therefore, we subsequently investigated the effects of MA on epigenetic modifications by assessing the histone acetylation and DNA methylation markers, H3K9ac and 5mC, respectively. Immunofluorescence (IF) staining revealed increased immunoreactivity of the H3K9ac and decreased 5 mC intensity in the MA-SCNT group in comparison to the Con-SCNT group (Figures 2B,C). Furthermore, we examined the mRNA expression of several genes (*BRG1, TET-3, Nanog, OCT-4, C-Myc* and *p300*) associated with epigenetic modification via PCR. As depicted in Figure 2D, the relative mRNA expression of these genes increased in the MA-SCNT compared to the control group. These results suggest that MA has a positive impact on EGA and epigenetic modification in cloned bovine embryos.

### Effects of MA on TERT, stem cell and inner cell mass (ICM) of bovine cloned blastocysts

**FIGURE 3:**
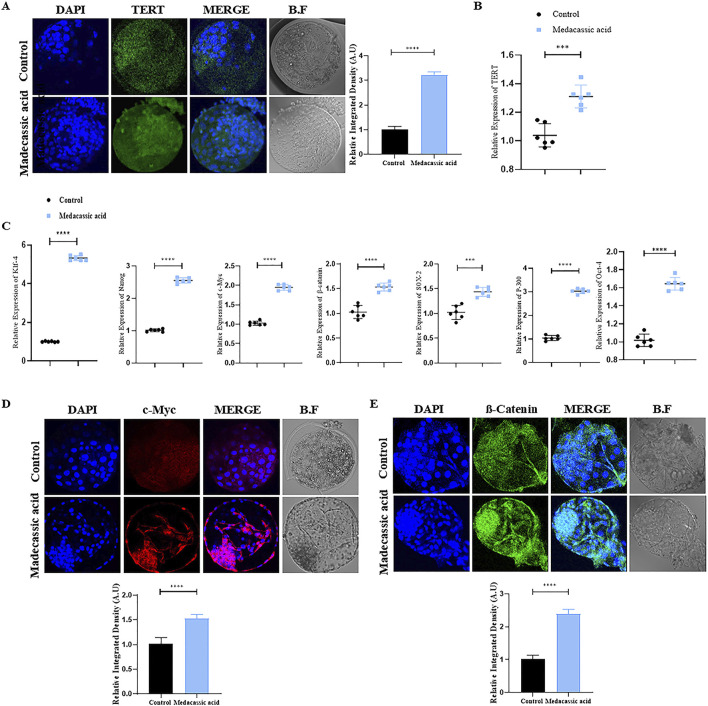
Effect of MA on stem cells related genes in the bovine cloned embryos. **(A)** Immunofluorescence labeling was used to assess the TERT protein level in the control and MA groups. FITC (green). **(B)** Analysis of mRNA expression of the TERT gene via qPCR. **(C)** mRNA expressions of Klf-4, NANOG, c-Myc, β-catenin, sox-2, P-300, and Oct-4 genes were analyzed in control and MA group. **(D,E)** Immunofluorescence shows the protein levels of c-Myc and β-catenin in the control and MA-treated groups. TRITC (red), FITC (green). The data are displayed as mean ± SEM. Bright field is represented by BF, and significant differences are shown by ***p ≤ 0.001, and ****p ≤ 0.0001. SEM stands for standard error of the mean.

Immunofluorescence (IF) staining was performed initially on all the tested bovine cloned blastocysts to analyze TERT protein expression. The result showed that MA significantly enhanced TERT protein levels in MA-SCNT-derived blastocysts compared to the control group (Figure 3A). Additionally, to ascertain the effect of MA on TERT gene regulation in cloned bovine blastocysts, quantitative reverse transcription polymerase chain reaction (RT-qPCR) was performed. The data revealed enhanced relative TERT mRNA expression levels in MA-SCNT blastocysts compared with those in the Con-SCNT group (Figure 3B). To further analyze the quality of bovine clone blastocysts, the relative expression of stem cells related genes was examined. All MA-SCNT blastocysts displayed significantly higher mRNA expression levels of the genes such as *klf-4*, *Nanog*, *SOX-2*, *OCT-4*, and *p-300* than those of the Con-SCNT group (Figure 3C). Moreover, the protein expression of c-Myc, a key ICM protein, observed via IF was also elevated following treatment with MA (Figure 3D). Likewise, MA significantly increased the relative levels of β-catenin compared to the Con-SCNT group as evident from IF staining (Figure 3E).

### Effects of MA on mitochondrial health, apoptosis, and implantation potential of SCNT-Derived blastocysts

**FIGURE 4:**
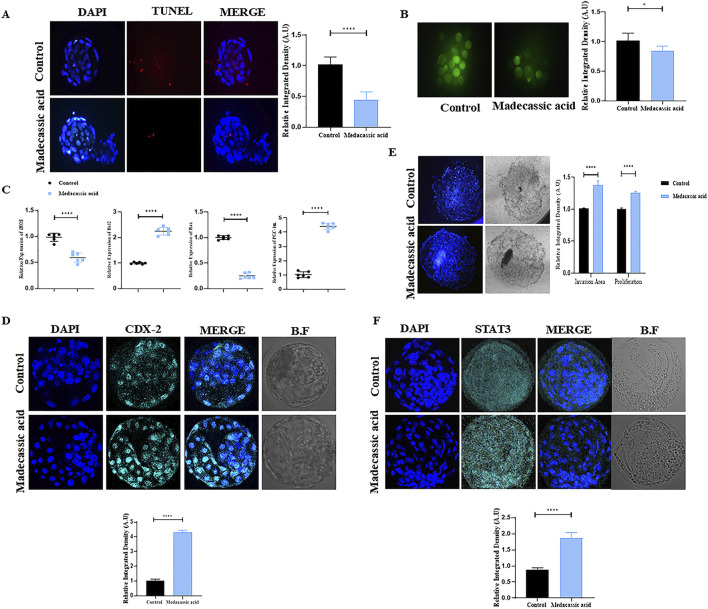
Effect of MA on apoptosis, mitochondria health, implantation potential and quality of bovine cloned embryo. **(A)** TUNEL assay of the day-8 SCNT-derived blastocysts in the control and MA group (n = 15 blastocysts per group). **(B)** ROS detection via H2DCFDA staining in control and MA-treated embryos. **(C)** Relative mRNA expression level of iNOS, BCL-2, Bax, and PGC1-α genes. For mRNA analysis a total of six blastocytes were used in triplicates. **(D)** The protein level of CDX-2 was examined via immunofluorescence staining in control and treated group. Alexa Flour 647 (cyan). **(E)** The invasion area and cell proliferation of implanted blastocysts in the control and MA group (Three blastocyst per group). show the implantation potential of the BL using Invasive assay. **(F)** Representative immunofluorescence images of STAT3 in the control and treatment groups. Alexa Flour 647 (cyan). The mean ± SEM is used to display the data. Significant differences are indicated by *p ≤ 0.005 and ****p ≤ 0.0001. Bright field is referred to as BF. SEM for standard error of the mean.

Medacassic acid exposure reduced cell apoptosis, as evidenced by the lower number of apoptotic cells in the MA-SCNT group compared with that in the control group (Figure 4A). Furthermore, MA improved mitochondrial integrity of SCNT-cloned blastocysts by reducing intracellular reactive oxygen species (ROS) levels, as revealed by DCF fluorescence microscopy (Figure 4B). Additionally, the mRNA expression of genes including *iNOS*, *Bcl-2*, *Bax*, and *PGC-1*, which are associated with mitochondrial function, was analyzed. The expression of *iNOS* and *Bax* was decreased following MA treatment, whereas that of *Bcl-2* and *PGC-1A* was increased compared with that in the control group (Figure 4C). Moreover, the IF analysis showed that the cloned blastocysts treated with MA revealed significantly higher CDX2 immunoreactivity compared to the Con-SCNT group, signifying improved the quality of the BL in the treated group (Figure 4D). An invasion assay was performed to further validate the implantation potential. The result showed that MA-SCNT blastocysts covered a greater invasion area and had more cells than the Con-SCNT-derived embryos, highlighting their implantation capacity (Figure 4E). Stat3 also plays a critical role in embryo implantation (Yu et al., 2022). The immunofluorescence analysis displayed elevated protein expression of STAT3 in the MA-SCNT group compared with those in the control group (Figure 4F). Overall, our data indicated that MA improves mitochondrial functions in blastocysts, inhibits cell death, and enhances the efficiency of embryo implantation.

### Molecular docking analysis of bTERT protein with MA

**FIGURE 5:**
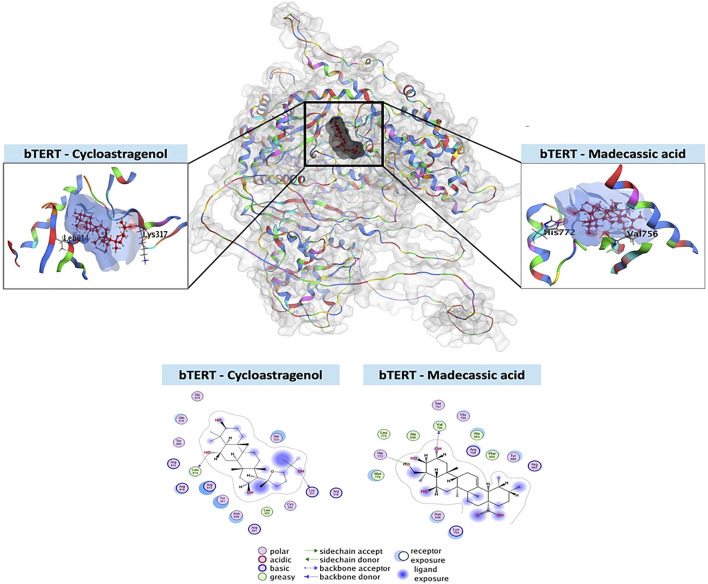
Molecular docking of MA with bTERT. The three-dimensional configuration of the test ligand (MA) and reference ligand (cycloastragenol) in the binding pocket of bTERT is shown in this 3D depiction. The two-dimensional arrangement of the ligands in the bTERT binding pocket, showing how each ligand’s atoms and amino acid residues interact molecularly. It shows the many kinds of interactions together with information on ligand exposure, acceptor/donor, polarity, acidic/basic, and greasy or neutral ligand residues.

In this study, bTERT was selected as the target protein for molecular docking with cycloastragenol (used as reference) and the test ligand MA. The docking score for MA shows better bond as well as more interaction between bTERT and MA as compared to the reference (Supplementary Table S2; Figure 5). The docking analysis revealed that cycloastragenol made two hydrogen bonds with the Lys317 and Leu614 amino acid residues of bTERT. On the hand, MA also makes two hydrogen bonds but different residues, i.e., Val756 and His772 amino acid residues of bTERT (Supplementary Table S2; Figure 5). On the whole, MA lower score, indicating better binding to bTERT than the cycloastragenol, making it a promising ligand.

### Molecular dynamics (MD) simulation analysis of bTERT protein with MA

**FIGURE 6:**
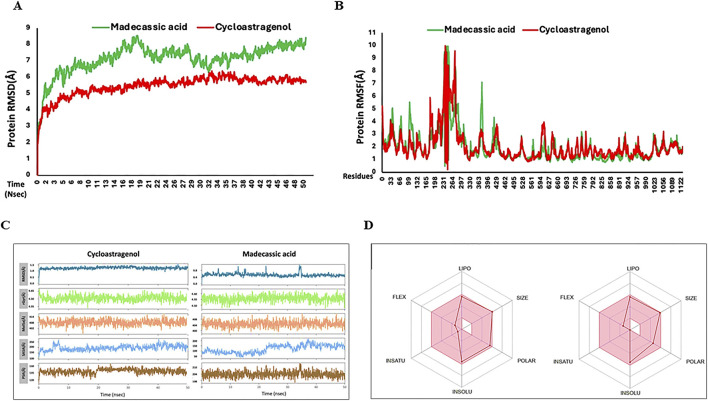
Root means square deviation (RMSD) and root mean square fluctuation (RMSF) values recorded during simulation and Ligand properties and oral bioavailability of ligands recorded during MD simulation. **(A)** Shown is the RMSD values of the backbone atoms of reference Cycloastragenol (reference, red color) and test MA (test, green color) ligand-protein complexes. **(B)** Root mean (RMSF) values for the test (green) and reference (red) ligand-proteins are shown throughout the simulation. **(C)** The ligand atoms' radius of gyration (rGyr), polar surface area (PSA), solvent accessible surface area (SASA), molecular surface area (MolSA), and root means square deviation (RMSD). **(D)** The color zones show the physiochemical space for oral bioavailability (retrieved from swissADME). LIPO (lipophilicity): −0.7<XLOGP3<+5.0; SIZE:<500 g/mol; POLAR (Polarity): 20Å^2^ <TPSA<130Å^2^; INSOLU (Insolubility) −6<LogS (ESOL) < 0; INSATU (Instauration): 0.25 < fraction Csp3<1; FLEX (Flexibility): 0<Num. rotatable bonds <9.

Recently, MD simulations have gained substantial importance in molecular biology and drug discovery. MD simulations have proven instrumental in the complete understanding of the atomistic energetics and mechanics of ligand protein binding, its stability, flexibility and ligand properties (Elkarhat et al., 2022; Hollingsworth and Dror, 2018). After molecular docking, the protein–ligand complex was subjected to MD simulations. RMSD is a key metric in MD trajectory analysis, indicating conformational stability (Han et al., 2022). In the bTERT-cycloastragenol complex, the RMSD value started at ∼ 3Å and stabilized at ∼5Å during the 50 ns run. For the bTERT–MA complex, the RMSD value started at ∼4Å and stabilized at ∼7-8 Å, indicating that the reference complex was slightly more stable than the test complex. Similarly, the ligand RMSD for the test was also slightly lower than that of the test ligand and it stabilized at ∼6Å and 7Å, respectively (Figure 6A). RMSF analysis is used for the observation of conformational shifts in residues during the MD simulation (Hu et al., 2023). Both the reference and test protein-ligand complexes displayed almost similar RMSF values, indicating similar flexibilities (Figure 6B). The ligand RMSD values reflect the stability of ligands throughout MD simulations. Observing the RMSD during simulations, it was found that MA has a lower RMSD as compared to the reference, indicating it is a more stable ligand during simulation (Figure 6C). The compactness of the complexes, which impacts rigidity, was assessed through the radius of gyration (rGyr). Higher rGyr values suggest higher instability, while lower values indicate enhanced stability (Ghahremanian et al., 2022). Over the simulation period, the rGyr reference was around 4.5Å while 4.55 Å for MA, indicating both have almost similar rGyr values (Figure 6C). Utilizing the probe radius of 1.4Å, similar van der Waals surface area of a water molecule, the molecular surface area (MolSA) was calculated. MolSA values fluctuated between 408 and 410Å for the reference ligand and between 404 and 408Å for MA, indicating MA-hTERS complex is slightly more stable. Solvent accessible surface area (SASA) values were higher for the reference ligand as compared to MA, showing favorable SASA values test ligand. Polar surface area (PSA) analysis showed that the reference ligand has lower scores than the test Madecassic acid ligand (Figure 6C).

Finally, the oral bioavailability of the reference and test ligands was examined using the SwissADME online database (Ranjith & Ravikumar, 2019). Both compounds exhibited good oral bioavailability (Figure 6D). Absorption, distribution, metabolism, excretion, and toxicity (ADMET) criteria are crucial for drug candidates. Given that ADMET-related problems account for the failure of more than 50% of medicines, these attributes are crucial for therapeutic development. In the present study, we conducted *in silico* ADMET evaluations using the online pkCSM application (Supplementary Table S3).

## Discussion

It has been widely accepted that most SCNT embryos exhibit numerous cellular and molecular abnormalities, including mitochondrial dysfunction, atypical epigenetic changes, altered gene expression, and deficiencies in telomere elongation (Huang et al., 2023; Kang et al., 2024). Due to the various manipulations during the SCNT procedure, such as serum starvation of donor cells, enucleation, electrofusion, and artificial activation, the developmental competence of the cloned embryos is lowered (Agarwal et al., 2006; Uhm et al., 2011). Shortening of telomere length and reduction in telomerase activity are major drawbacks of SCNT-cloned embryos (Kalmbach et al., 2014; Kong et al., 2014; Nadri et al., 2022). Previous studies show a relationship between the initial telomere length of cloned embryos and the telomere length in granulosa cells (used as a donor). This implies that the cloned embryo’s telomere length may be influenced by the donor cell’s telomere length, Also the serum starvation condition also significantly decreases the telomerase activity in the donor cell. (Betts et al., 2001; Jiang et al., 2025). Here, we investigated the effects of madecassic acid (MA), a natural telomerase activator, on the quality and developmental potential of SCNT-derived cloned bovine embryos. Our findings demonstrate that MA significantly enhances telomerase activity, embryo cleavage rates, blastocyst formation, and the expression of genes critical for embryonic development, offering a promising approach to improving SCNT outcomes.

Telomere shortening is a hallmark of SCNT embryos and a primary cause of chromosomal instability (King et al., 2006). In normal embryonic development, telomerase is activated during the zygotic stage to restore telomere length, but this process is often defective in cloned embryos, leading to impaired genome stability (Schaetzlein and Rudolph, 2004; Zhao et al., 2014). The telomerase reverse transcriptase (TERT) subunit is essential for maintaining telomere length and regulating chromatin remodeling, which supports embryonic genome activation (EGA) and early development (Xu and Yang, 2000). Furthermore, telomerase regulates the expression of DUX-4 by remodeling the chromatin and promoting zygotic genome activation (Zhang et al., 2023). Our results show that MA treatment significantly increases telomerase activity in granulosa cells and cloned embryos, correlating with improved telomere maintenance. This enhancement likely contributes to the observed increase in cleavage and blastocyst rates, as longer telomeres support rapid cell division during early embryogenesis (Kong et al., 2014). Furthermore, MA upregulates the expression of EGA-associated genes, including NFYA, SP1, DPRX, GSC, ß-catenin, DUX, and ARGFX, suggesting that its telomerase-activating properties facilitate robust embryonic genome activation.

Epigenetic reprogramming is another critical challenge in SCNT, as incomplete reprogramming of the somatic nucleus often results in aberrant DNA methylation and histone modifications (Niemann, 2016). In SCNT-cloned embryos, donor cells maintain a high level of methylation, which leads to improper epigenetic modifications (Planello et al., 2014). Epigenetic defects have been observed in the early development of cloned bovine embryos (Han et al., 2003). Cloned embryos frequently exhibit elevated global DNA methylation (5 mC) and reduced histone acetylation (e.g., H3K9ac), which hinders pluripotency and embryonic development (Chen et al., 2022).

Our data reveals that MA-treated SCNT embryos display higher global H3K9ac and lower 5 mC levels, indicating improved epigenetic reprogramming. These changes are likely mediated by TERT, which influences chromatin accessibility and histone modifications in telomeric and sub-telomeric regions (Blasco, 2007; Song and Johnson, 2018). By promoting an open chromatin state due to telomerase activity (Marión and Blasco, 2010), MA enhances the pluripotency of cloned embryos, as evidenced by the upregulated expression of key pluripotency markers such as OCT4, NANOG, SOX2, KLF4, p300, and c-Myc. These transcription factors are essential for maintaining the inner cell mass (ICM) and ensuring blastocyst quality, further supporting the developmental benefits of MA (Ballini et al., 2019).

Abortion due to the unsuccessful implantation of embryos is a major problem in animal cloning (Edwards, 2006; Young, 2003). The implantation potential of SCNT embryos is often compromised due to defective trophoblast development and insufficient telomere maintenance, leading to high rates of pregnancy loss (Stewart et al., 2006). Long telomeres and active telomerase activity are crucial for the rapid cell cycling required in trophectoderm cells, which form the placenta (Yang et al., 2008). Active telomerases maintain telomere length and enhance embryonic development (Ozturk et al., 2014). TERT plays direct and indirect roles in genes related to ICM and pluripotency (Liu et al., 2007). TERT increases the expression of important transcription factors that preserve pluripotency in ESCs (Teichroeb et al., 2016). To support the activation of pluripotency, TERT directly interacts with OCT4 and SOX2 to stabilize their transcriptional complexes (Yeo and Ng, 2013). Wakayama shows that telomere length can be proper maintain during 25 generation of serial cloning (Wakayama et al., 2013). Same result was reported by (Bermejo-Alvarez et al., 2010). TERT ensures effective reprogramming and self-renewal by facilitating the binding of NANOG and KLF4 to target promoters (Li et al., 2020). Previous studies have shown that TERT is linked to genes related to the ICM, and cloned embryos often show altered expression of genes related to the ICM (Galan et al., 2010; Khan et al., 2023; Kotian et al., 2024; Xu et al., 2021). Our findings reveal that MA enhances TERT expression and telomerase activity, improving trophoblast gene expression in treated embryos. Assisted reproductive technology (ART) is associated with altered telomerase activity, likely due to increased mitochondrial dysfunction, oxidative stress, and epigenetic modifications (Zacchini et al., 2019). We therefore examined the effect of MA on blastocysts' apoptosis using a TUNEL assay. Our finding revealed that the MA treatment reduce the apoptosis. This suggests that MA not only improves early embryonic development but also enhances implantation potential, potentially increasing pregnancy success rates. In drug design and discovery, molecular docking studies play a crucial role in understanding ligand-protein interactions. This simulation technique effectively utilizes energy minimization and binding energy assessments to reveal the interactions between drug molecules and their target proteins (Topal et al., 2021). In silico analyses further support MA’s efficacy, demonstrating strong binding affinity to bovine TERT (bTERT) compared to the reference ligand cycloastragenol. Cycloastragenol is natural compound and well known for the TERT activation (Idrees et al., 2023; Khan et al., 2023). Molecular dynamics simulations confirmed the stability and compactness of the MA-bTERT complex, while ADMET studies indicated favorable pharmacological properties, reinforcing MA’s potential as a therapeutic agent for SCNT.

In summary, MA addresses multiple barriers to SCNT efficiency by enhancing telomerase activity, improving epigenetic reprogramming, upregulating pluripotency and EGA-associated genes, and supporting trophoblast/embryo development. These improvements translate into higher cleavage and blastocyst rates, better embryo quality, and potentially greater implantation success. By mitigating the molecular defects inherent to SCNT, MA represents a novel and effective strategy for advancing cloning technology. Future studies should explore the long-term effects of MA on post-implantation development and offspring health to validate its utility in reproductive biotechnology.

## Conclusion

We found that the activation of telomerase upon MA treatment improves the development and implantation potential of bovine SCNT-cloned embryos. Furthermore, MA’s natural telomerase activity can probably normalize the epigenetic modifications that are mostly altered in SCNT-cloned bovine embryos. Moreover, our data also revealed that telomerase activation via MA enhances the invasion capability of cloned embryos, which can result in a successful pregnancy.

## Data Availability

The original contributions presented in the study are included in the article/Supplementary Material, further inquiries can be directed to the corresponding authors.

## Supplementary material

SUPPLEMENTARY FIGURE 1 The table show the optimal concentration of IVF embryos development.

SUPPLEMENTARY FIGURE 2 The table shows the Hydrogen bond between the reference protein and bTERT of the Medacassic acid.

SUPPLEMENTARY FIGURE 3 The table show the Absorption, distribution, metabolism, excretion, and Toxicity (ADMET) criteria.
